# Cell encapsulation in gelatin methacryloyl bioinks impairs microscale diffusion properties

**DOI:** 10.3389/fbioe.2023.1193970

**Published:** 2023-08-31

**Authors:** Elvan Dogan, Christina Holshue, Anant Bhusal, Roshni Shukla, Amir K. Miri

**Affiliations:** ^1^ Advanced Biofabrication Laboratory, Department of Biomedical Engineering, New Jersey Institute of Technology, Newark, NJ, United States; ^2^ Department of Mechanical Engineering, Rowan University, Glassboro, NJ, United States; ^3^ Department of Mechanical and Industrial Engineering, New Jersey Institute of Technology, Newark, NJ, United States

**Keywords:** mass transport, diffusivity, gelatin methacryloyl, bioprinting, bioink

## Abstract

Light-assisted bioprinted gelatin methacryloyl (GelMA) constructs have been used for cell-laden microtissues and organoids. GelMA can be loaded by desired cells, which can regulate the biophysical properties of bioprinted constructs. We study how the degree of methacrylation (MA degree), GelMA mass concentration, and cell density change mass transport properties. We introduce a fluorescent-microscopy-based method of biotransport testing with improved sensitivity compared to the traditional particle tracking methods. The diffusion capacity of GelMA with a higher MA significantly decreased compared to a lower MA. Opposed to a steady range of linear elastic moduli, the diffusion coefficient in GelMA varied when cell densities ranged from 0 to 10 × 10^6^ cells/ml. A comparative study of different cell sizes showed a higher diffusivity coefficient for the case of larger cells. The results of this study can help bioengineers and scientists to better control the biotransport characteristics in light-assisted bioprinted microtissues and organoids.

## 1 Introduction

A large class of bioinks is three-dimensional (3D) water-saturated polymer networks in hydrogels. Natural or synthetic polymeric networks in hydrogels provide high water content (typically between 70% and 99% of volume), time-dependent or viscoelastic behavior, tunable degradability, cell-friendly environment, and adaptable crosslinking for tissue engineering and regenerative medicine (Axpe et al., 2019). The fluid-saturated hydrogels provide drug delivery mechanisms and offer a biomimetic modeling platform (Caló and Khutoryanskiy, 2015). Recent evolutions in the biofabrication of hydrogel-based bioinks allow for making tunable biomechanics and structural heterogeneity required for modeling biomedical systems (Unagolla and Jayasuriya, 2020; Dogan et al., 2021). A key aspect is to replicate the biotransport properties of biomedical systems.

The structural heterogeneity of cell-laden hydrogels regulates their solute biotransport across the matrix, including the transport of oxygen and nutrients through entangled pores (Novosel et al., 2011). There are two dominant biotransport mechanisms to deliver biological agents to any specific organ or target tissue: *diffusion* and *convection* (Shkilnyy et al., 2012; Ray et al., 2019). Convection is regulated by flow properties such as velocity and requires a driving force such as fluid pressure gradient. Diffusion is driven by gradients of physiologically relevant agents (i.e., nutrients, growth factors, signaling molecules, drugs), which serve as a governing mechanism in hydrogel scaffolds/systems (Ramanujan et al., 2002). The diffusion rate of proteins, nutrients, ions, and oxygen impact cell growth, proliferation, and biological functionality (Leddy et al., 2004; Miri et al., 2018; Jin et al., 2019; Hassan et al., 2021). Quantitative analysis of the diffusion rates will allow scientists and bioengineers to design optimum scaffolds for desired functionalities.

Hydrodynamic, free volume, and obstruction theories are the prevalent models used to explain and predict the biotransport properties in fluid-filled systems (Amsden, 1998; Axpe et al., 2019). In the hydrodynamic theory, the particle motion is governed by the surrounding fluid flow (i.e., convection-dominated). It is primarily due to the medium of the solute or particles, viscous drag forces and the fluid flow pattern. The hydrodynamic theory also relates diffusivity to the size and shape of the particles, in addition to the fluid properties. This theory assumes that there are no significant interactions among the moving particles (Amsden, 1998; Byron Bird et al., 2006). In free volume theory, there is enough space for particles to move without obstacles and the transport is determined by the concentration and size of such free spaces. The movement capacity increases with an expansion of the free volume fraction, as it provides more space for the free movement of the particles. This theory has often been applied in the study of polymer diffusion and gas permeation through membranes (Vrentas and Duda, 1978; Ramesh et al., 2011). In the case of obstruction theory, physical barriers, boundaries, or complex structures within the material are considered. The theory suggests that the diffusivity drops with increased interactions and collisions between the particles and obstacles (Mackie et al., 1997; Axpe et al., 2019). Compared to the previous theories, obstruction theory is a better choice for biotransport in non-disperse networks, such as colloidal suspensions and biological systems. In this work, the obstruction theory (assuming no convection) was selected to analyze experimental data.

The diffusivity (or hydraulic permeability) can be a design criterion in the biofabrication of microtissue models (Bhusal et al., 2022) and tissue engineering scaffolds (Dogan et al., 2020). It depends on several factors, including hydrogel concentration, composition, and the size of the molecules or particles. Researchers have developed customized testing methods such as fluorescence recovery after photobleaching (FRAP), diffusion chambers, or transwell assays to measure the diffusivity or hydraulic permeability of hydrogels. For example, Leddy et al. (2004) used fibrin, gelatin, and alginate gels to assess the diffusion coefficients of fluorescently active dextran, as the transport agent, via FRAP (loaded with human adipose-derived stem cells: 10^7^ cells/ml). Shkilnyy et al. reported the apparent diffusion coefficients of RhD-B and FITC-bovine serum albumin (BSA) in fibrin gel (4 mg/ml) with cell encapsulation. They performed fluorescence imaging to produce a calibration curve for the release signal. After obtaining the intensity graph, they calibrated this data with the concentration gradient of the agent inside the fibrin gel (Shkilnyy et al., 2012). Axpe et al. (2019) has developed a multiscale diffusion model combining three common theoretical frameworks in the literature. McCarty et al. measured the specific hydraulic permeability of Matrigel™ at selected mass concentrations (1% and 2% v/v) under varying perfusion pressures (ranging from 0 to 100 mmHg). The results showed that 2% Matrigel™ had a lower permeability and higher stiffness than 1% Matrigel™. Their permeability values aligned with the predictions of the fiber matrix model (McCarty and Johnson, 2007). Miri et al. introduced a customized spherical indentation-based testing method to estimate the hydraulic permeability of gelatin methacryloyl (GelMA) for a wide range of mass concentrations and crosslinking conditions (Miri et al., 2018). Numerical simulations and Biot’s theory of poroelasticity were combined to simulate fluid transport within the gel. The hydraulic permeability ranged from approximately 0.002–1 μm^2^/Pas.

Dromel et al. studied the relationship between the water fraction in hydrogels and their physical, chemical, and diffusion properties, for enhanced drug delivery to retinal regeneration (Dromel et al., 2021). They compared the diffusion and release of human epidermal growth factors within different injectable hydrogels: gelatin-hydroxyphenyl propionic acid and hyaluronic acid-tyramine-based hydrogels. Using theoretical diffusion models, hydrodynamic modeling was found to be efficient for the measured solute diffusion coefficient. Shenoy and Rosenblatt reported BSA and dextran diffusivity in (30 mg/ml) collagen as D_37°C_ = 2.2 × 10^−7^ cm^2^/s for BSA, and D_37°C_ = 2.0 × 10^−7^ cm^2^/s for 69 kD dextran (Shenoy and Rosenblatt, 1995). In another study, Shkilnyy et al. compared the diffusivity of rhodamine B (RhD-B) and FITC-BSA in fibrin gel (4 mg/ml) in which the diffusion coefficient of RhD-B was found to be 3.43 ± 0.25 × 10^−6^ cm^2^/s while for FITC-BSA it was found to be 0.18 ± 0.25 × 10^−6^ cm^2^/s (Shkilnyy et al., 2012). Despite the efforts to measure the diffusivity response, the role of cell density in cell-laden gels has been assumed to be negligible by many researchers. This may hamper the prediction accuracy of the gel diffusivity in cell-laden models for drug delivery and other similar applications.

In this work, we presented a simple method to study the permeability-structure relations in GelMA based on varying biofabrication conditions. We selected GelMA scaffold (Bhise et al., 2016), which is a semi-synthetic, biodegradable, photo-crosslinkable, biocompatible hydrogel system (Ruedinger et al., 2015; Pepelanova et al., 2018). The mechanical properties of GelMA, such as permeability, stiffness, and degradation time, can be tailored through bioinks and/or bioprinting parameters (Kuo et al., 2016; Miri et al., 2018; Wang et al., 2018) These parameters include I) the degree of methacrylation (MA degree), II) the intensity of photo-crosslinking, III) the light exposure time, and IV) the mass concentration (Miri et al., 2018). Although different cell types might contribute to light absorbance rate and solute diffusivity, the tumor cells are selected as being used in microtissues and organoid models (Dogan et al., 2022). To consider the role of cell morphology in our biotransport data, we chose soft tissue sarcoma cells (HT-1080), breast tumor cells (MDA-MB-231), and human mammary epithelial cells (HMEC) for comparison. Our preliminary data and previously published paper showed that 5%–10% GelMA provides a stiffness value between ∼1 and ∼15 kPa (Miri et al., 2018). Soft tissue tumors are heterogeneous in terms of both anatomic and inter-patient stiffness and perfusion (Stylianopoulos et al., 2018). The mean stiffness of soft tissue sarcoma has been reported to be around 2.37 ± 1.49 kPa (i.e., 0.89–6.3 kPa) (Pepin et al., 2019). We conducted customized and conventional experiments to measure the bulk diffusivity and compression modulus of GelMA constructs with different cell densities. This study will help bioengineers develop more predictable cell-laden GelMA models.

## 2 Materials and methods

### 2.1 Cell preparation

Human fibrosarcoma cells (HT-1080; ATCC; Manassas, VA) were cultured in the appropriate growth media recommended by ATCC. All the cells were passaged according to standard practices, in which they showed >80% confluency for each passage and were seeded at desired concentrations for our experiments. All chemicals, media, and substrates were mainly purchased from VWR (Radnor, PA), with some exceptions mentioned in the text. HT-1080 cells were cultured at 37°C with 5% CO2. Dulbecco’s Modified Eagle Medium (DMEM, VWR, Radnor, PA) was mixed with 10% v/v FBS and 1% v/v Pen/Strep to feed cells every 2 days. Required cell density for experiments was collected after trypsinization using 1 mL of trypsin-EDTA solution (0.25% Corning, Manassas, VA) centrifuged (Beckman Coulter, Avanti J-15R) for 3 min at 900 rpm and 4°C for the experimental setup.

In addition to HT-1080, epithelial, human breast cancer cells (MDA-MB-231) and human mammary epithelial cells (HMEC; ATCC; Manassas, VA) were also cultured in appropriate growth media recommended by ATCC. After trypsinizing, cells were suspended in DPBS with varying cell densities (0.1–10 × 10^6^ cells/ml) and homogeneously pipetted. The samples were loaded in a 96-well plate (Storage Plate 96-Well Flat Bottom Ltd., New York, NY) for light absorbance analysis to observe the effect of cell type on the cross-linking. The data were recorded at a multi-well scanning spectrophotometer using a wavelength of 490 nm.

### 2.2 Bioink preparation

**FIGURE 1:**
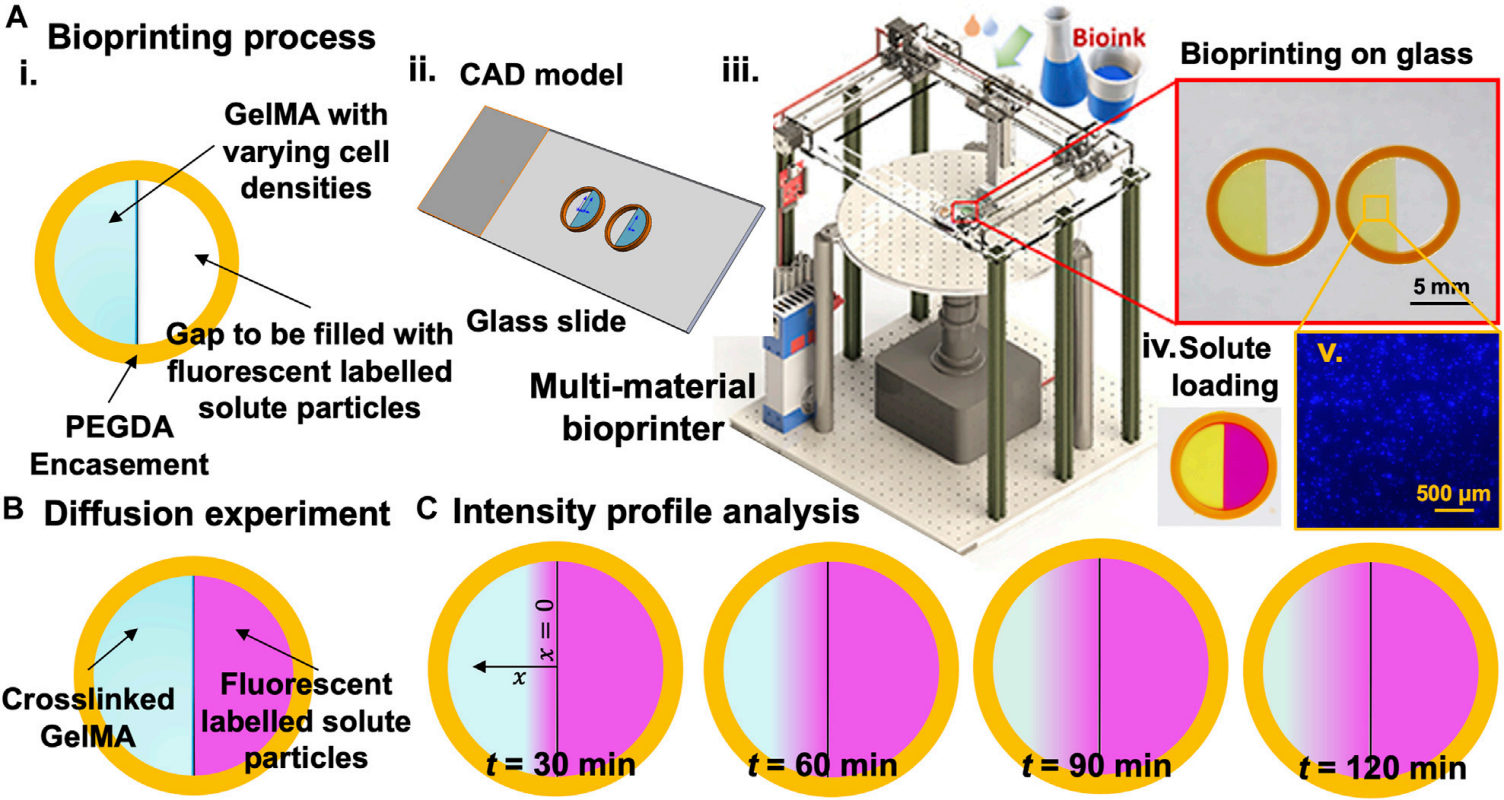
The experimental approach for measuring 1D concentration gradient through hydrogel samples within printed constructs with high control over size and shape. **(A).** Bioprinting process: i. schematic view of the bioprinted sample, ii. CAD model of two samples on a glass slide, iii. custom-made multi-material DLP bioprinter and bioprinted samples, iv. Samples filled with fluorescent solutes, and iv. An example of the cell distribution (10^6^ cells/ml). **(B).** Schematic of samples to be used for intensity profile analysis under the fluorescent microscope. **(C)**. Intensity readings at different time points to be used for the diffusion profile analysis.

GelMA was synthesized according to an established protocol (Van Den Bulcke et al., 2000). In a 100 mL glass flask of Dulbecco’s phosphate-buffered saline (DPBS; Sigma-Aldrich, St. Louis, MO) and 10% w/v Porcine Skin Gelatin (CAS Number 9000-70-8; Sigma-Aldrich, St. Louis, MO) were mixed. The flask was covered to prevent evaporation and was stirred using a magnetic stir bar on a hot plate at 60°C until fully dissolved (approximately an hour). Following gelatin dissolution in the DPBS, methacrylic anhydride at 3 ml and 8 ml were slowly pipetted into the solution to obtain “low MA” and “high MA,” respectively. The temperature was reduced to 50°C, and the solution was stirred and allowed to react for an hour. Pre-warmed DPBS was 5x the volume of the initial solution and was added to the solution after an hour to stop the reaction. Dialysis tubing (12–14 kDa cut-off molecular weight) was used to seal the solution. The dialysis tubes were then submerged in DI water for a week at 40°C. After a week, the solution from the dialysis tube was collected into a glass flask and freeze-dried until the synthesized GelMA demonstrated a porous foam structure. GelMA solution (5, 7, and 10% w/v) was prepared with DPBS and pre-warmed at 40°C with constant stirring. Once fully dissolved and homogenized, a final concentration of 0.07% w/v lithium phenyl-2,4,6-trimethyl-benzoyl-phosphinate (LAP; Sigma Aldrich) was used as the photo-initiator. After sterilizing using 0.2 µm filters, GelMA solutions were prepared at the desired concentration and agitated to allow cells to disperse through the GelMA volume. The orange color in bioprinted samples (Figure 1A) was used for better visualization here and to demonstrate our simple geometry. Photoabsorber was excluded from this study to minimize any potential counter-effects.

### 2.3 ^1^H nuclear magnetic resonance (NMR)

The degree of methacryloyl functionalization was quantified by using ^1^H NMR according to the previously described method (Hoch et al., 2013; Li et al., 2016). ^1^H NMR spectra were collected using an NMR spectrometer, Bruker AVIII-500-MHz equipped with RT TXI probe (H1, C13, N15), B008T (Bruker, Billerica, MA) at a frequency of 500 MHz. Before the measurement, 20 mg of GelMA macromers was completely dissolved in 1 mL deuterium oxide containing 0.05% w/v 3-(trimethylsilyl)-propionic-2,2,3,3-d4-acid sodium salt for calibration (Sigma-Aldrich, St. Louis, MO, United States). Data was processed using Bruker TopSpin 3.6.5 software (Bruker, Billerica, MA). The methacryloyl substitution was quantified using the following equation (see Supplementary Table S1; Supplementary Figure S1):

$$Degree\;of\;functionalization\;\left(DoF\right)=1-\left(lysine\;methylene\;proton\;of\;GelMA,\delta =2.9\;ppm\right)/\left(lysine\;methylene\;proton\;of\;gelatin,\delta =2.9\;ppm\right)\times 100\%.$$ (1)

### 2.4 Bioprinting process

The bioprinting applies a digital micromirror device (Texas Instrument, Dallas, TX) with an ultraviolet (UV) light system (Visitech; Wetzlar, Germany) of wavelength ∼380 nm and light intensity of ∼0.7 W/cm^2^ (at the focal plane) to print hydrogels (Bhusal et al., 2021). The platform was equipped with a tri-axial stage to move the printing platform and a rotational movement to rotate the Petri dish platform controlled by a custom-written program. The fabrication process starts with layer-by-layer slicing. Then, the bioink is kept in the UV-grade Petri dish, and the printing process is initiated. The layer thickness and exposure time were selected as 50 μm and 2 s for each layer (Bhusal et al., 2021). The printing platform then moves to the printing position, and a linear z-stage lowers the platform. The program turns on the UV light, and the first layer is cured. The platform then moves to the second layer position, and the bioink flows inside between the first layer and the base of the reservoir to print the second layer. The process was used to ensure the uniformity of the samples.

### 2.5 Unconfined compression

Standard compression testing was performed to determine the material’s stiffness under physical load. We followed an established protocol (Schuurman et al., 2013) and made disk-shape samples of 10 mm in diameter and 5 mm in height. We analyzed the elastic moduli for different hydrogel sets (Table 1, *n* = 4) to evaluate the roles of mass concentration (5, 7, and 10% w/v), MA degree (low and high), and cell density (0–10 × 10^6^ cells/ml) in gel stiffness. The samples were placed between the flat metal plates of the mechanical tester (Model 5848; Instron Inc., Norwood, MA) using a 10 N load cell, and the tests were performed using the displacement rate of 1 mm/min (or strain rate of 20% per min). The rate was selected high enough to minimize the effects of hydrogel relaxation. The elastic modulus was calculated as the slope of the stress-strain curve from up to 8%–10% strain (i.e., almost linear range).

**TABLE 1:** Diffusion coefficient of RhD-B and the elastic modulus of GeIMA for two different MA degrees and varying cell concentrations.

Bioink MA degree	Bioink Mass concentration	Cell density x10^6^ cells/ml	Bulk diffusion coefficient (µm^2^/s)	Bulk elastic modulus (kPa)
Low	5% w/v	0	554.278 ± 7.184	1.633 ± 0.522
	7% w/v	0	510.481 ± 6.824	2.577 ± 0.536
		0.1	517.623 ± 6.025	2.775 ± 0.500
		0.5	501.632 ± 2.729	2.768 ± 0.241
		1	398.839 ± 10.18	2.372 ± 0.425
		3	372.320 ± 8.630	2.229 ± 0.435
		5	351.328 ± 5.992	2.147 ± 0.541
		8	389.470 ± 19.00	1.689 ± 0.459
		10	463.413 ± 19.07	1.847 ± 0.240
	10% w/v	0	422.888 ± 5.170	6.895 ± 1.217
High	5% w/v	0	311.044 ± 8.545	2.913 ± 0.426
	7% w/v	0	284.349 ± 12.26	9.673 ± 1.014
		0.1	302.090 ± 12.97	n/a
		0.5	307.994 ± 9.053	n/a
		1	279.160 ± 14.09	n/a
		3	251.737 ± 8.094	n/a
		5	243.903 ± 5.816	n/a
		8	336.251 ± 21.45	n/a
		10	411.188 ± 12.06	n/a
	10% w/v	0	255.754 ± 8.020	15.454 ± 0.657

### 2.6 Diffusion experiment

Diffusion experiments were conducted to assess the diffusion coefficient of RhD-B in GelMA. We evaluated the apparent diffusion coefficients of the RhD-B (Sigma-Aldrich) agent in crosslinked bioinks (*n* ≥ 4) with varying cell density (0–10 × 10^6^ cells/ml, see the example in Figures 1A–v) through the relative intensity method using the Nikon Eclipse Ti2 microscope and analyzed according to Fick’s second law (Shkilnyy et al., 2012). A 1.2 × 10^−5^ M RhD-B solution was prepared after several trials with different RhD-B concentrations to obtain a smooth intensity profile in GelMA samples. 3D bioprinted samples (Bhusal et al., 2021) for diffusion experiment consisted of a polyethylene glycol (PEGDA) circular frame in 8 mm inner and 10 mm outer diameter and a half-cylindrical GelMA construct 8 mm in diameter and 2 mm thick, as shown in Figure 1B. After, we printed a half inner GelMA core, placed it under a Nikon fluorescent microscope, and filled the half gap with RhD-B solution. The intensity profile was recorded for 30, 60, 90, and 120 min during the experiment with an exposure time of 30 ms. To take an intensity reading, a straight line of 4 mm was drawn in the Nikon software from the edge of the GelMA, which meets the RhD-B towards the center of the GelMA sample. Experiments were performed in triplicate (*n* = 3). The intensity profiles along the line were exported, and these steps were repeated for all the samples to gather initial and interval readings. The raw data were imported into Excel, turned into calibration curves, and processed to evaluate the results (Supplementary Figures S2 and S3). Diffusion coefficients (µm^2^/s) (Caló and Khutoryanskiy, 2015) for RhD-B were obtained by fitting concentration gradient data using Fick’s second law Eq. 7.

The fluorescent properties of the selected agent allowed for the visualization of mass transfer into the GelMA porous structure (Figure 1C). We assumed mediums to be homogeneous, and the fluorescence signal is linearly related to the concentration of the RhD-B particles. Fick’s second law (1D) is applicable to model the diffusion of the particles:

$$\frac{\partial c}{\partial t}=D\frac{\partial^{2}c}{\partial x^{2}}$$ (2)

In Eq. 2, *c* is the solute concentration, *x* is distance, *t* is time, and *D* is the diffusion coefficient of the structure. The variable x represents the perpendicular distance measured from the center of our hydrogel sample. In this case, the center corresponds to the center of the circle, where the edge of the hydrogel is adjacent to the RhD-B solution. The distance extends from this edge to infinity, which in our printed sample, measures 4 mm (radius). When *x* equals zero x=0 , it indicates the edge of the hydrogel, as shown in Figure 1C. As our experiments start with a tracer concentration of zero within the gel sample, and the tracer is introduced from the source at x=0 , with a constant concentration of $c_{o}$ , we have applied the following boundary conditions 3):

$$c\left(x=0,t\right)=c_{o}\;\;c\left(x,t=0\right)=0\;\;c\left(x=\infty ,t\right)=0$$ (3)

To solve Fick’s second law, the error function equation and error function complement equation were used as shown below:

$$\mathrm{erf}\left(z\right)=\frac{2}{\sqrt{\pi }}\int_{z}^{\infty }e^{{-t}^{2}}dt$$ (4)

$$\mathrm{erf}c\left(z\right)=1-\mathrm{erf}\left(z\right)$$ (5)

$$z=\frac{x}{2\sqrt{Dt}}$$ (6)

$$C\left(x,t\right)=Co\;*\;\mathrm{erf}c\left(z\right)$$ (7)

$$\frac{C\left(x,t\right)}{Co}=\mathrm{erf}c\left(z\right)$$ (8)

We used the above formulation to translate our intensity readings into diffusivity (Supplementary Figures S2 and S3) and calculate the apparent diffusion coefficients of RhD-B. According to Einstein’s equation of Brownian motion (Einstein, 1905) and semi-empirical tortuous flow in porous media equations (Koponen et al., 1996), the effective diffusion coefficient through hydrogels $\left(D_{eff}\right)$ , can be expressed as a function of a structure factor, p , the porosity, φ , and bulk solution diffusion coefficient, $D_{0}$

$$D_{eff}=\frac{D_{0}}{1+p\left(1-\varphi \right)}$$ (9)

### 2.7 Release study

We conducted an established release experiment to validate our approach for the diffusion experiments (Ma et al., 2018). Hydrogel precursor solutions were prepared in DPBS, with a final concentration of 5%–10% w/v for high- and low-MA GelMA, 0.07% w/v for LAP, and a final concentration of 0.1 mM RhD-B. Disk-shape samples of 10 mm in diameter and 5 mm in height were made, and we conducted a couple of trials to optimize RhD-B concentration and ratio of DPBS to sample amount. RhD-B-loaded hydrogels were immersed in 3 mL DPBS by shaking at 200 rpm and 37 °C. At different times, the release medium (1 mL) was removed and replaced with an equal volume of DPBS. RhD-B concentration was quantified by SpectraMax i3 (Molecular Devices, San Jose, CA) at 485/535 nm. Seven readings from each well were collected and averaged. The total amount of RhD-B was calculated by a standard curve of RhD-B in DPBS versus fluorescence intensity (Supplementary Figure S4). The release experiments were performed in triplicate (*n* = 3).

### 2.8 Biological assays

The viability of encapsulated cells and spheroids in GelMA was assessed using one Live/Dead assay (PromoCell GmbH, Heidelberg, Germany). At endpoints, the samples were rinsed with PBS (Sigma-Aldrich), incubated for 40 min with Calcein-AM (1 mM; live cells in green) and ethidium homodimer 1 (6 mM; dead cells in red), and then rinsed again. Encapsulated cells in GelMA were then imaged with a fluorescence microscope (Nikon, Melville, NY) through FITC and TRITC filters. The combined Live/Dead images were processed in ImageJ software to estimate the percentages of live and dead cells.

### 2.9 Statistical analysis

Statistical analysis was performed in GraphPad software (GraphPad Software, Inc., San Diego, CA) statistical tool. A one-way analysis of variance (one-way-ANOVA) and two-way analysis of variance (two-way-ANOVA) tests were used for data analysis. A value of *p* < 0.05 was considered statistically significant. Finally, a linear correlation analysis was performed among the governing parameters using Excel (Microsoft).

## 3 Results and discussions

### 3.1 MA functionalization in GelMA

The conjugation of methacryloyl to the gelatin molecules was confirmed by the ^1^H NMR spectra (see Supplementary Figure S1). The increased signal at δ = 5.4 and 5.7 ppm (i.e., the protons of the methacrylate vinyl group) and decreased signal at δ = 2.9 ppm (i.e., the protons of the methylene of lysine signal) confirmed the rate of modification degree with the MA (Li et al., 2016). The intensity of the proton signal of the aromatic amino acid moieties in gelatin was used to normalize the intensity of other protons in various samples because their signal stayed constant over time. As a result, the DoF was determined by comparing the proton signal of GelMA and unmodified gelatin at δ = 2.9 ppm. By adjusting the feed ratio of MA to gelatin, the DoF of the three types of GelMA macromers was found to be 50.14% ± 2.04% for low-MA, 65.32% ± 2.06% for high-MA (see Supplementary Table S1). Increased MA is associated with the introduction of more crosslinking sites that expand the connectivity within the network. As a result, the MA factor provides control over the hydrogel’s mechanical and biological properties (Zhu et al., 2019). ^1^H NMR analysis validation shows the efficiency of our protocol in preparing GelMA samples and a baseline for biotransport analysis.

### 3.2 Stiffness of cell-laden GelMA

**FIGURE 2:**
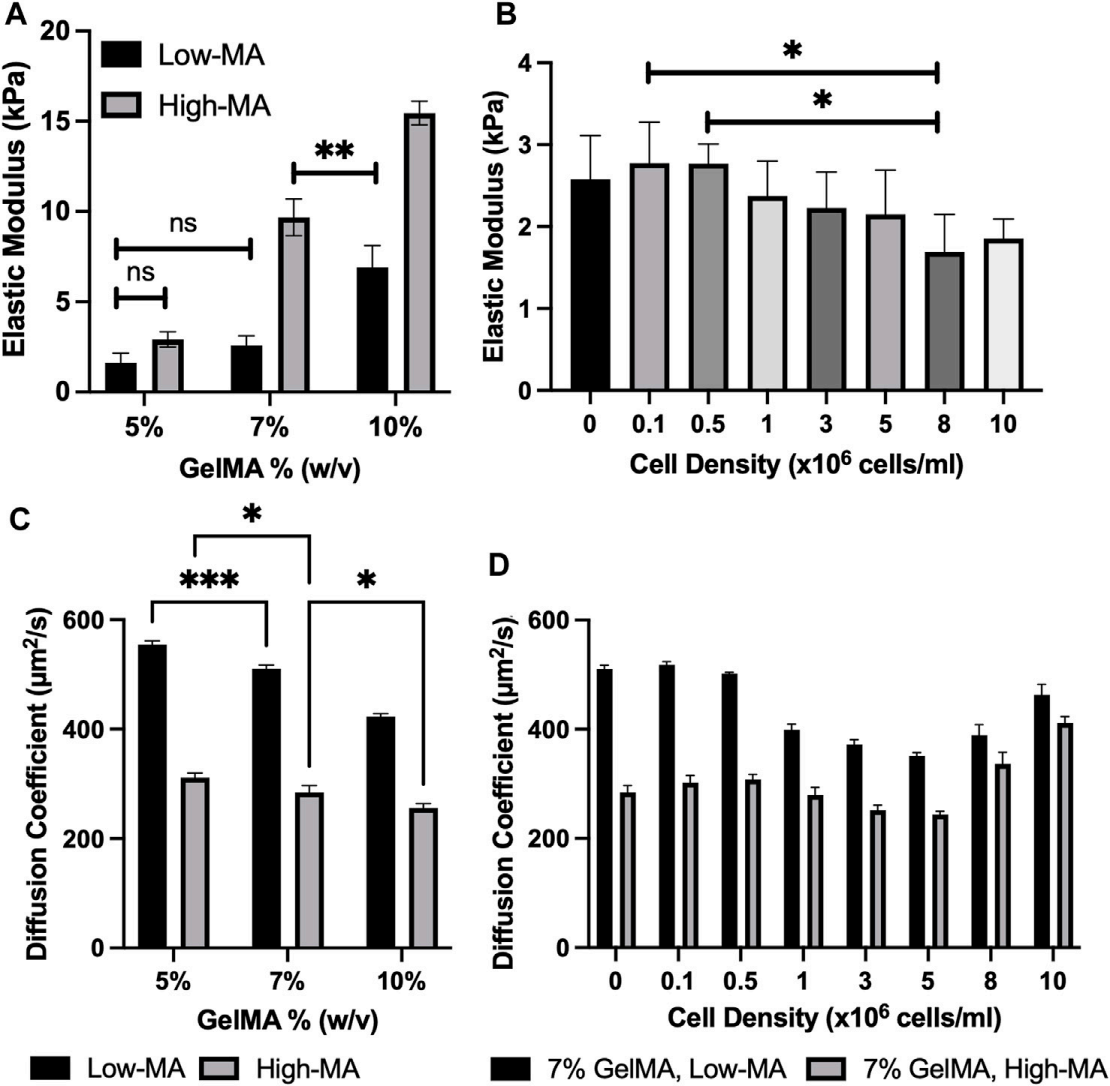
**(A)**. Elastic modulus for 5%, 7%, and 10% w/v GelMA at low, medium, and high MA. All conditions were significantly different (***p < 0.001) unless indicated; **(B)**. Elastic modulus for cell-laden GelMA (7%: low-MA) with varying cell concentrations. All conditions were non-significantly different for cell-laden GelMA samples unless indicated (ns *p* > 0.05, *p ≤ 0.05, **p ≤ 0.01,***p ≤ 0.001). All data are presented as the mean ± SD (n = 4); **(C)**. Apparent diffusion coefficients (μm^2^/s) of RhD-B in 5, 7, and 10 GelMA (w/v) with Low and High MA degrees. All conditions were significantly different (***p ≤ 0.001) unless indicated; **(D)**. Apparent diffusion coefficients (μm^2^/s) of cell-laden 7% GelMA with low and high-MA degrees (ns *p* > 0.05, *p ≤ 0.05, **p ≤ 0.01, ***p ≤ 0.001). All data are presented as the mean ± SD (*n* = 3).

The unconfined compression testing of GelMA disk samples with different MA degrees and mass concentrations was used to quantify the relationship between the stiffness and cell density. The trend can also be used to understand the transport properties of GelMA. We selected 7% low MA for simplicity and evaluated the elastic moduli of crosslinked samples (*n* ≥ 4) with a cell density of 0–10 × 10^6^ cells/ml. Figure 2A shows the elastic modulus for GelMA 5%, 7%, and 10% w/v at low and high MA. The elastic modulus was found to be ranging from 1.633 ± 0.522 kPa for 5% w/v low-MA GelMA to 15.454 ± 0.657 kPA for 10% w/v high-MA GelMA. The cell encapsulation led to an increased level of stiffness (although not statistically) and then lower values at high cell densities, such as 10 × 10^6^ cells/ml (*p* < 0.01), while MA degree and GelMA concentration had a significant impact on stiffness, as shown in Figures 2A, B. This would support the notion that a lower photo-crosslinking density can occur in the presence of large cell volumes. In summary, the cell density insignificantly impacts the hydrogel stiffness after bioprinting.

The unconfined compression testing has been used to measure the modulus of soft samples in the field. The linear modulus may not be sensitive enough to accurately capture the effect of cell density. The cell presence may contribute to the material resistance after the linear deformation (i.e., when the network chains are deformed to their limits). There might be other reasons for the lack of sensitivity in such testing methods. For instance, the mechanism involves load transfer through the interconnections of the fiber cross-sections, providing an additional contribution to the reinforcement process (Castilho et al., 2018).

### 3.3 Biotransport properties of cell-laden GelMA

The fluorescent properties of the selected agent allowed for visualization of mass transfer into the GelMA porous structure. We assumed mediums to be homogeneous, and the fluorescence signal is linearly related to the concentration of the RhD-B particles (validated by our preliminary tests). We used Eqs 5–9 to translate our diffusion experiments into numbers (see the examples in Supplementary Figure S3). The average diffusion coefficient of the agent through GelMA was higher at lower mass concentrations and low MA, as shown in Figures 2C, D; Table 1. The diffusivity of RhD-B in high-MA samples with varying GelMA concentrations demonstrates a low significance level. The diffusivity is different in low-MA samples with varying GelMA concentrations (Figure 2C). Interestingly, these results indicate that the high-MA case dominates over GelMA concentration in terms of forming a dense and less diverse hydrogel network. The higher value means a larger pore size or ease of fluid movement within the microstructure. We also tested 7% GelMA loaded by cells in a 1–10 × 10^6^ cells/ml range. At a cell density of 5 × 10^6^ cells/ml, the diffusion coefficient decreases ∼33% for low MA compared to acellular GelMA. From 5 × 10^6^ to 10 × 10^6^ cells/ml, an increase in the diffusion coefficient up to ∼31% was observed in Figures 2C, D; Table 1. Light reduction can occur due to high cell density, which decreases the degree of GelMA crosslinking. A similar pattern in high-MA GelMA was also observed. The MA factor impacts the degree of photo-crosslinking; thus, high- and low-MA cases were used to test the role of cell densities for the physical properties of GelMA 7% samples. Three different mass concentrations of GelMA with and without the cell presence were used: 5%, 7%, and 10% GelMA. It was observed that the average diffusion coefficient of the agent was higher at lower mass concentrations.

**FIGURE 3:**
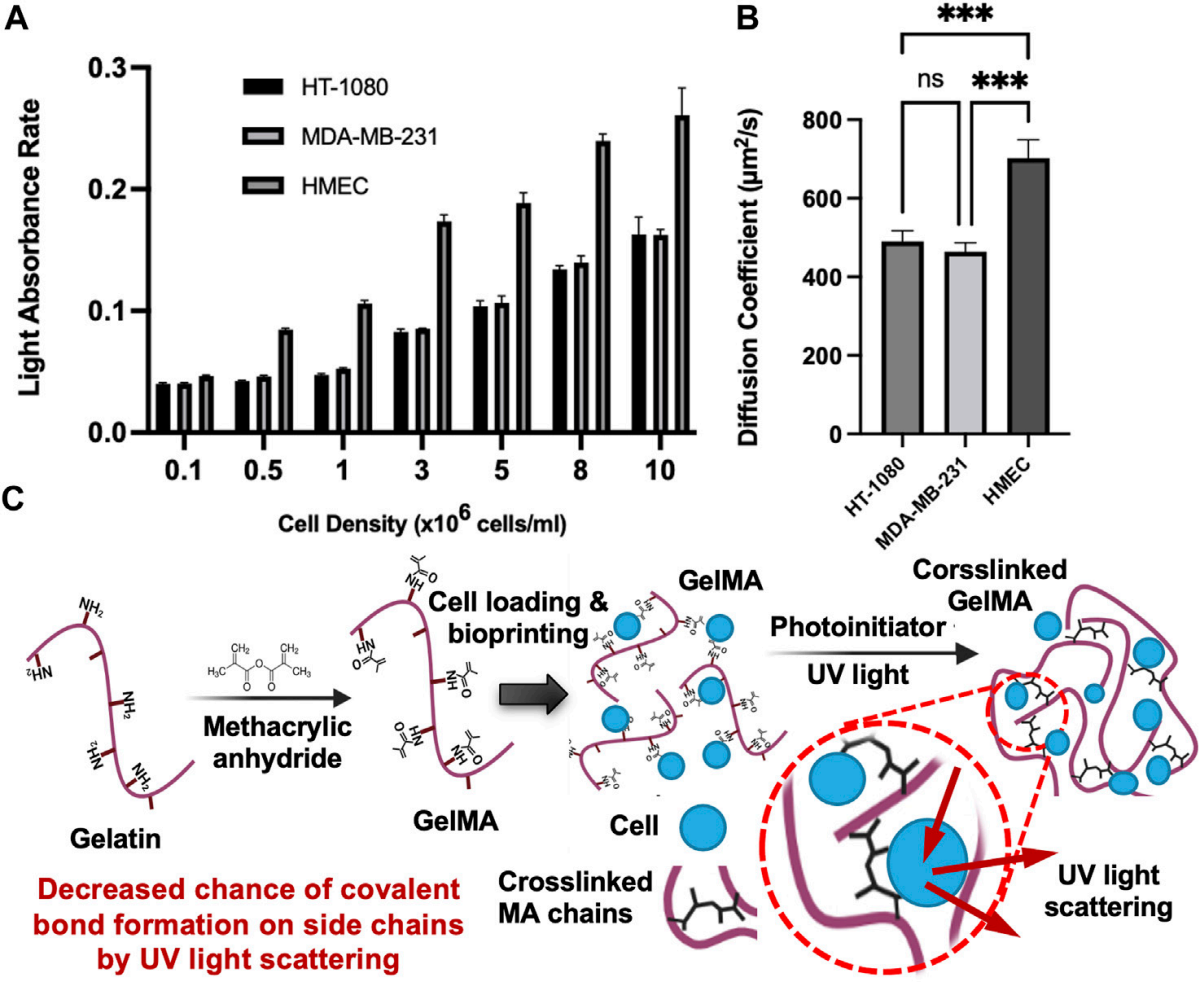
Light absorbance rate and diffusion coefficients for 3 cell types **(A)**. Light absorbance rate of HT-1080, MDA-MB-231, and HMEC with varying cell densities (0.1 million to 10 million). All conditions were significantly different (***p ≤ 0.001) unless indicated; **(B)**. Diffusion coefficients of RhD-B in HT-1080, MDA-MB-231, and HMEC encapsulated in 7% w/v GelMA samples with 10 million cell density (ns: *p* > 0.05, *p ≤ 0.05, **p ≤ 0.01,***p ≤ 0.001). All data are presented as the mean ± SD (n = 3); **(C)**. Proposed mechanisms for increased diffusivity coefficient in crosslinked GelMA with high cell densities.

A summary of the diffusion coefficients and elastic moduli is shown in Table 1. The trend of the diffusion coefficient is significant when compared to diverse cell densities indicating a possible interference in the photo-crosslinking through absorption of light energy and possible inhibition by oxygen-based bioproducts around the cells (oxygen is a major inhibitor of our crosslinking). The authors speculate that cell pellets in the hydrogel precursor absorb light through the membrane (which is practically challenging to validate). Cell membranes, mostly of lipids and proteins, have refractive indices in the range of 1.46–1.54 where the refractive index of the suspending medium is assumed to be 1.33 (Meyer, 1979). The higher cell density raises the overall refractive index of the hydrogel. A higher refractive index means a slower traveling speed of light and an increased change in the direction of the light (or less focus of light energy). From this perspective, by an increase in cell density, we may anticipate a consistent reduction of the elastic modulus (as seen in Figure 2B) and an increase in the diffusion coefficient. To assess whether cell-light interaction is specific to the select phenotype, we compared the selected type with 3 cell types. The data in Figure 3A shows a dependency of light absorption capacity on the cellular type, being the highest in HEMC. This would suggest that the HMECs will lead to more diverse data considering their larger size (60–100 μm in diameter) (Romeo et al., 2006) when compared to HT1080 (10–15 μm in diameter) and MDA-MB-231 (8–2 µm in diameter) (TruongVo et al., 2017). Figure 3B demonstrates diffusion coefficients of RhD-B in HT-1080, MDA-MB-231, and HMEC encapsulated in 7% w/v GelMA samples with 10 million cell density. Our results demonstrated a direct correlation between cell size and light absorbance rate. Larger cell size can raise the solute diffusivity. This indicates an impaired crosslinking, which produced light absorption of larger cells and the hindrance for GelMA chain connectivity.

Another speculation about the role of the cell density is that the lower density causes pore-clogging and/or reduces the pore size of hydrogel, lowering the possibility of the diffusion of solute particles. This phenomenon can be explained by Brownian particle motion through porous structure decreases via more obstacles (Alsaid et al., 2021). Therefore, the impact of cell density on diffusivity can be inferred from Eq. 9 for the specified cell densities. On the other hand, we observed that high cell density increased solute diffusivity, which may be due to high cell density causing light scattering during photo-crosslinking that causes a reduction in crosslinking energy to form a covalent bond between methacrylate side chains. This is illustrated in Figure 3C. The lower crosslinking denotes a larger pore size, which acts against the cell-induced frictions or drags onto the fluid flow.

The release behavior represents the mass transport behavior of bioprinted hydrogel and is related to a large group of bioprinted models: drug delivery scaffolds. We used this established approach to validate our methodology. The results in Supplementary Figure S5 demonstrate a similar trend to the diffusivity results, indicating that the high-MA case dominates over GelMA concentration in terms of forming a dense hydrogel network. While low-MA 5% GelMA releases 83% of the loaded RhD-B, there is a decreasing trend with increased GelMA concentration, which shows a higher significance level compared to the high-MA samples with varying GelMA concentrations. The release test sensitivity is questionable compared to the diffusion test since spectrometers can only detect significant differences in a narrow range of concentrations. After reaching saturation levels of higher concentrations, it is difficult to detect a significant difference between higher molar concentrations of RhD-B for lower concentrations. The resolution and sensitivity of microplate readers are inadequate to monitor calcium flux in live cells compared to the fluorescent microscopy method (Meijer et al., 2014).

## 4 Concluding remarks

We conducted diffusivity and unconfined compression experiments for porous cell-laden hydrogel systems with tailored physical properties. The purpose of this study was to understand the role of MA, mass concentration, and cell density on the apparent mass transport properties in light-assisted bioprinted GelMA constructs. The stiffness and diffusion properties of cell-laden samples were tailored for desired biological applications (cell viability and cell distribution data shown in Supplementary Figures S6 and S7). The diffusion coefficients of RhD-B were found to be considerably affected by cell densities higher than 10^6^ cells/ml, a common range for biofabrication methods. This should be a deciding factor for creating cell-laden scaffolds or drug-delivery systems.

The results indicate the significant effect of MA on diffusivity, a factor neglected by many published reports. The diffusivity of RhD-B in high-MA samples with varying GelMA concentrations shows a low significance level. In contrast, the diffusivity in low-MA samples with varying GelMA concentrations (Figure 2C) exhibits significant differences. These results are intriguing as they suggest that the high-MA case dominates over the case of GelMA mass concentration in the formation of less diverse hydrogel networks, as demonstrated by the structural images in Supplementary Figure S8. This would denote a counter-effect of cell density and MA crosslinkers density on the diffusivity.

Lastly, the impaired biotransport properties can depend on the cell size and morphology, assuming a well-homogenized cell distribution (Supplementary Figure S7 and Supplementary Video S1). This can be less predictable when making multi-cellular models. The results of this study have implications for bioengineers and scientists in predicting the biotransport properties of gelatin-based constructs (Schwartz et al., 2020). The future steps will involve investigating the use of data modeling (or correlation analysis) for predicting the elastic modulus and diffusion coefficient of GelMA with varying fabrication parameters. This will require collecting a meaningfully higher number of biotransport experiments and mechanical testing to screen different variables beyond this proof-of-concept study. The perspective correlation models will be a game-changer for the field of biofabrication.

## Data Availability

The raw data supporting the conclusion of this article will be made available by the authors, without undue reservation.
