# Supplementary material for: Cell encapsulation in gelatin methacryloyl bioinks impairs microscale diffusion properties
Source: Front Bioeng Biotechnol. 2023 Aug 31;11:1193970. doi: 10.3389/fbioe.2023.1193970 (PMC10507472; doi:10.3389/fbioe.2023.1193970)
Supplement: Supplementary file 1 [file DataSheet1.pdf]

frontiers

Supplementary Material

Cell Encapsulation in Gelatin Methacryloyl Bioinks Impairs Microscale Diffusion Properties

Elvan Dogan, Christina Holshue, Anant Bhusal, Roshni Shukla, Amir K. Miri*

*** Correspondence:** Dr. Amir K. Miri: am3296@njit.edu, 973-596-6366

1 Supplementary Data

1.1 Scanning Electron Microscopy Imaging

The structure of the GelMA hydrogel was analyzed using a scanning electron microscope (SEM). The hydrogel samples were printed as described. Then samples were placed in an incubator in DPBS for 24h at 37 °C and swollen hydrogels were frozen (-80 °C) and subsequently lyophilized (n = 4) for each group. The lyophilized samples were cut and their cross-sections were coated with platinum using a turbo sputter coater (EMITECH, K575X). SEM images were acquired (JSM-7900F Schottky Field Emission Scanning Electron Microscope at a voltage of 5 kV.

1.2 Volumetric Cell Distribution

Volumetric cell distribution was imaged using the Nikon Eclipse Ti2 microscope using 4X magnification. Prior to encapsulation, the cells were stained with CellTracker™ Blue CMAC Dye (Thermo Scientific, Waltham, MA) according to the manufacturer's protocol. Densities of 0.1-3 x10^6 cells/ml were chosen for a case of GelMA 7% w/v. After the bioprinting process, z-stack images were obtained and processed to demonstrate volumetric cell distribution and 2D projection (maximum intensity) profiles.

2 Supplementary Figures and Tables

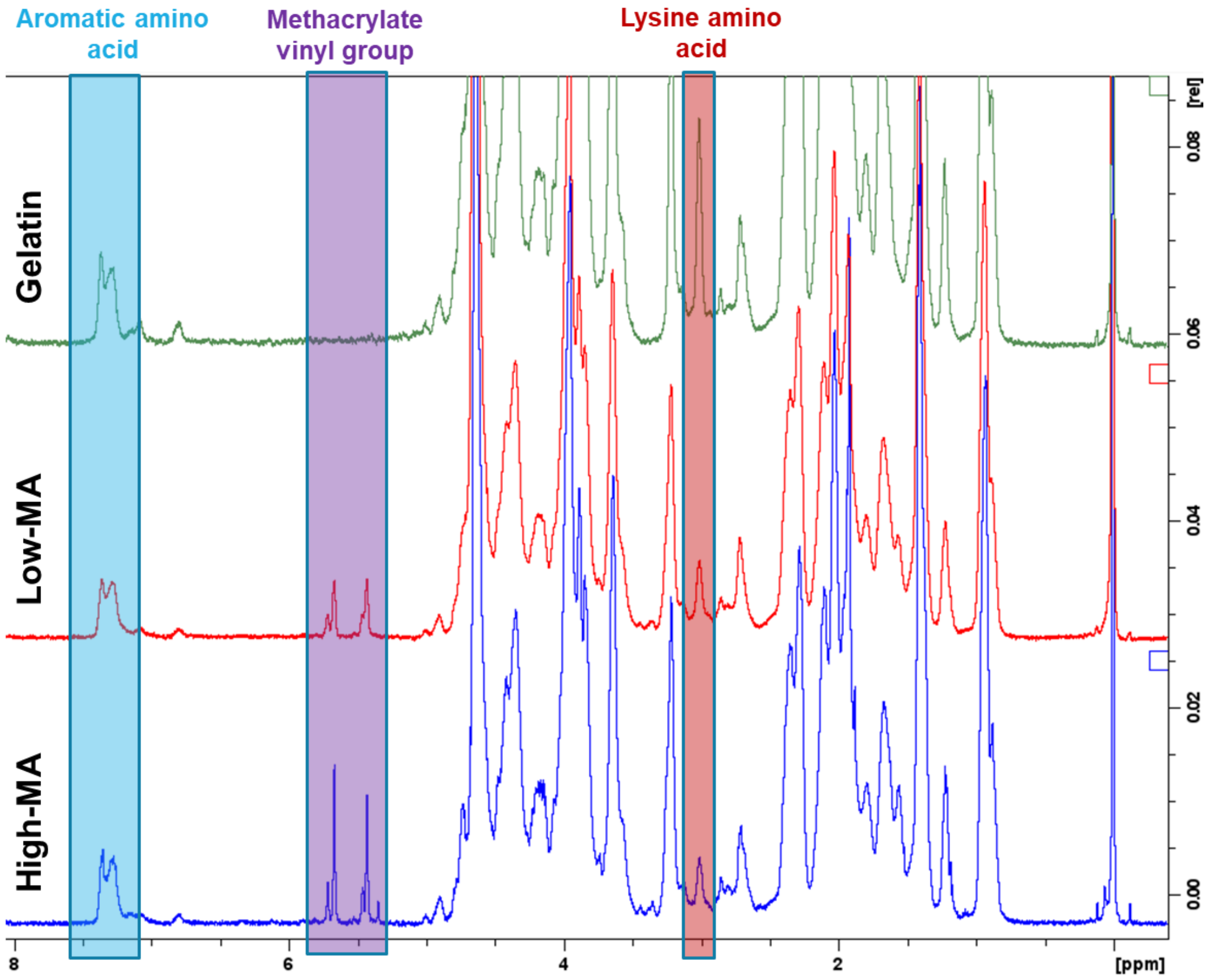

Figure S1. ^{1}H NMR spectra of gelatin and GelMA with different DoF of low-MA, and high-MA.

Table S1. The DoF (%) of GelMA with varying gelatin/MA ratios.

**Sample**	**Gelatin (g) / MA (ml)**	**DoF (%) ±**
Low MA	10/3	50.14 ± 2.04
High MA	10/8	65.32 ±2.06

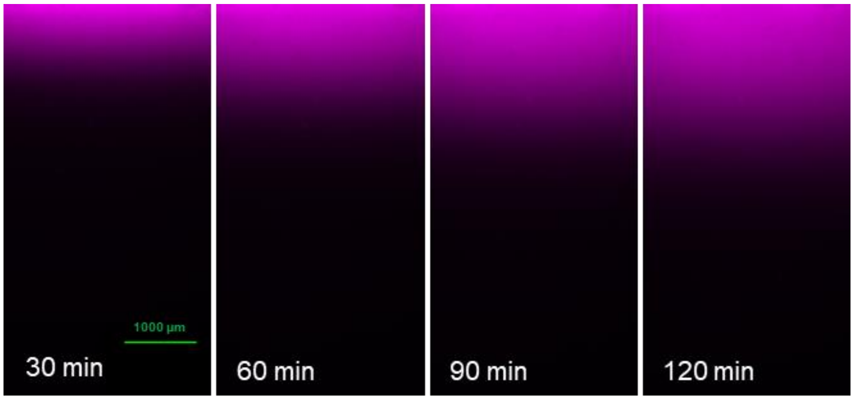

Figure S2. RhD-B Diffusion through GelMA

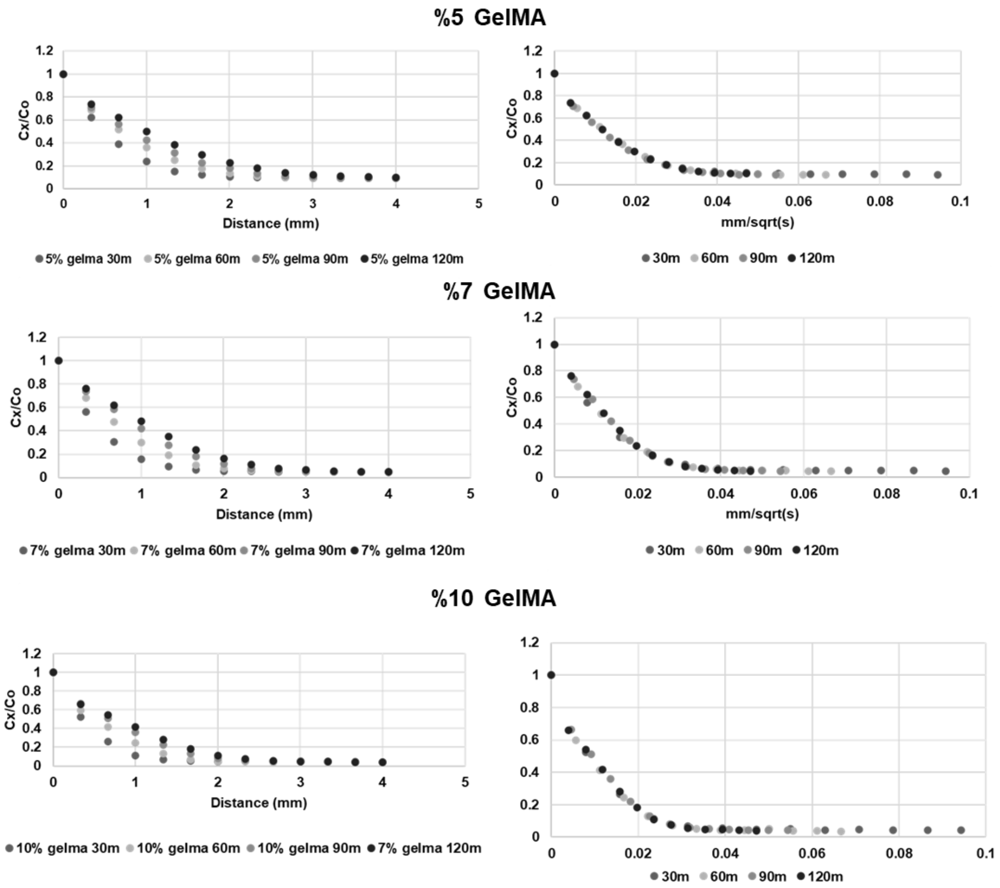

Figure S3. Mean concentration profiles of RhD-B in low MA GelMA with varying concentrations (5-10% w/v) at different time intervals. The left column is the concentration gradient, and the right column shows the diffusion profile of RhD-B.

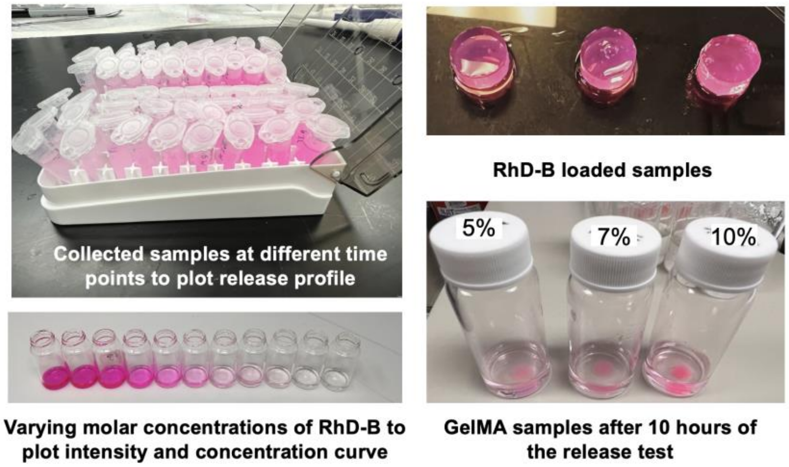

Figure S4. Release test conditions

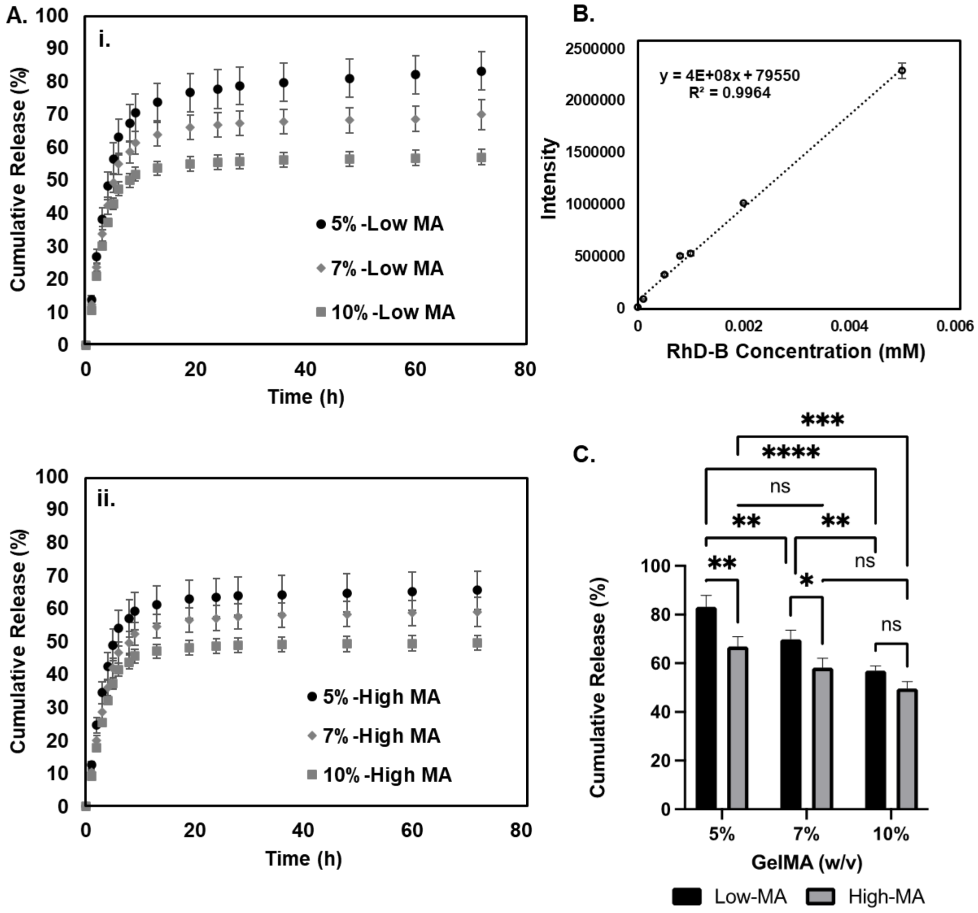

Figure S5. Release test results: Cumulative release profile of RhD-B loaded for two cases: **A.** Low Ma GelMA; and **B.** High Ma GelMA for 72 hours; **C.** Molar concentration and intensity calibration curve. **D.** Cumulative release rate in percentage at 72nd h (*ns* $P > 0.05$, $*P \le 0.05$, $**P \le 0.01$, $***P \le 0.001$). All data are presented as the mean ± SD (n = 3).

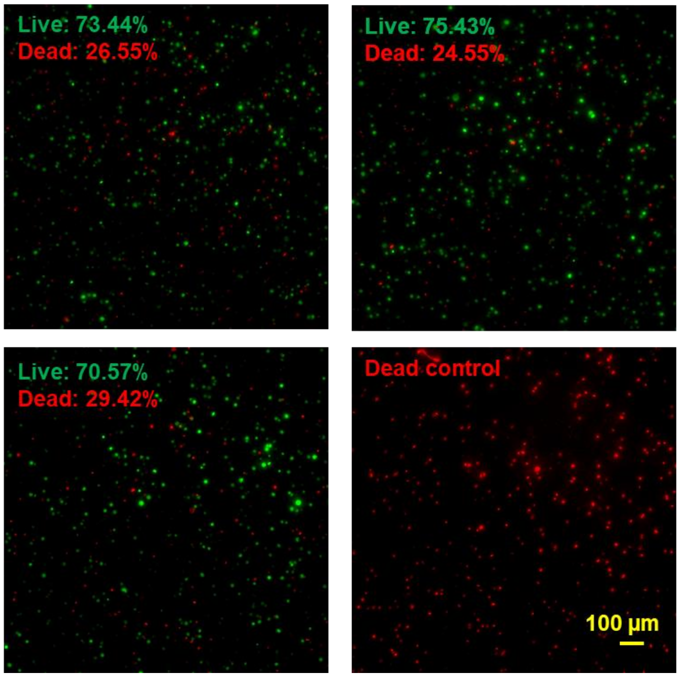

Figure S6. Live (green) - dead (red) images in printed GelMA samples

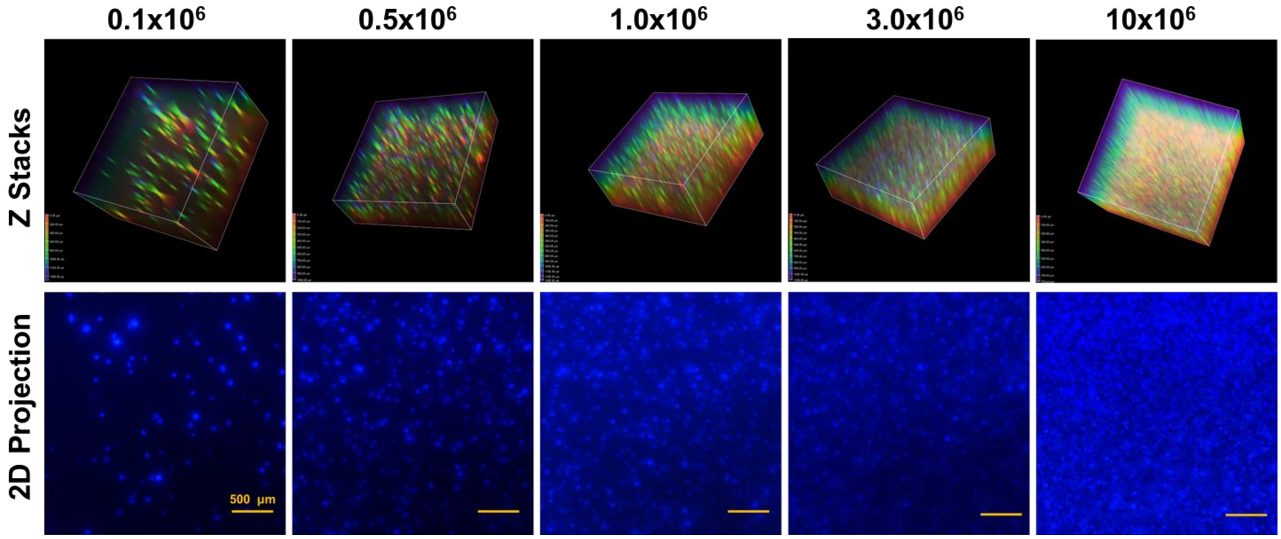

Figure S7. Cell distribution of bioprinted GelMA 7% w/v samples in 2D projection and volumetric cell distribution forms for selected cell densities (cells/ml).

https://drive.google.com/file/d/1IyopXKztHGhtVrmkDrdclIh68n0a54Nd/view?usp=sharing

Video S1. Cell distribution of bioprinted GelMA samples in volumetric cell distribution form. Cell density: 10^6 cells/ml.

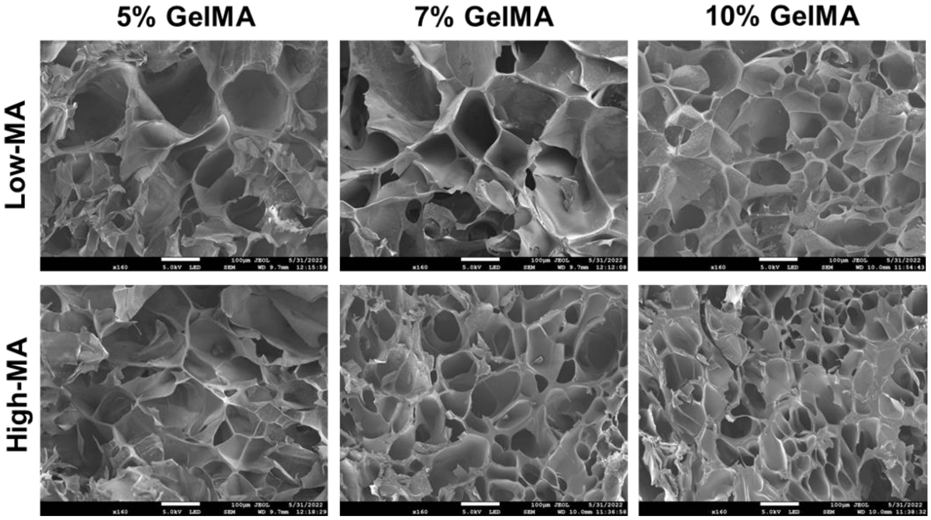

Figure S8. Porosity imaging for the three different concentrations (5, 7, and 10% w/v) and two different methacrylation degrees (low and high) through SEM, scale bar shows 100 µm.
